# Mobile Health: making the leap to research and clinics

**DOI:** 10.1038/s41746-021-00454-z

**Published:** 2021-05-14

**Authors:** Joy P. Ku, Ida Sim

**Affiliations:** 1 Department of Bioengineering, Stanford University, Stanford, CA USA; 2 Division of General Internal Medicine, University of California San Francisco, San Francisco, CA USA

**Keywords:** Translational research, Health care, Research data

## Abstract

Health applications for mobile and wearable devices continue to experience tremendous growth both in the commercial and research sectors, but their impact on healthcare has yet to be fully realized. This commentary introduces three articles in a special issue that provides guidance on how to successfully address translational barriers to bringing mobile health technologies into clinical research and care. We also discuss how the cross-organizational sharing of data, software, and other digital resources can lower such barriers and accelerate progress across mobile health.

## Introduction

Mobile devices have been a disruptive technology in many industries, but their impact on healthcare has yet to be fully realized. This is not due to a lack of interest. There are ~85,000 health apps^1,2^ available for download, and over $8 billion was invested in “digital health” in 2018^3^. Novel miniaturized sensors are being developed to continuously detect biomarkers (e.g., from sweat^4^, tear fluid^5^) that have traditionally been measured within a clinic. These developments are creating new possibilities for a vision of medicine that is more data-driven and personalized (e.g., ref. ^6^). In this article, we will refer to the use of such sensors and apps to collect personalized data for health in a ubiquitous manner as mobile health (mHealth).

As has been noted elsewhere^7,8^, data collection is only the first step in developing mHealth solutions that improve health outcomes. Clinicians and other stakeholders need to be convinced of the benefits of mHealth, and to-date it has been challenging to draw clear conclusions about the efficacy of these solutions, given the conflicting outcomes and heterogeneity in the implementation of mHealth interventions. This holds true whether assessing the impact of mHealth on hospital admission rates among patients with heart failure, adherence to prescribed rehabilitation exercises or lifestyle changes, or health outcomes like weight and blood pressure^9,10^. Myriad other factors, such as integration into the clinical or research study workflow, cost of implementation, usability of the device, and adequacy of privacy protections, also affect the likelihood of a solution transitioning from the prototype stage to routine use within research and clinics. Coming out of a multi-disciplinary workshop called mHealth Connect, three articles in this issue explicate some of these factors and provide guidance on how to successfully address translational barriers for different use cases. Specifically, the articles describe considerations when (1) selecting a suitable wearable sensor for a given application; (2) analyzing observational health behavior data generated by mHealth apps and devices; and (3) integrating these technologies into the clinical environment.

Their recommendations demonstrate the critical role data has in this new paradigm, so in addition to introducing the three articles, this paper calls for cross-organizational sharing of digital resources to accelerate progress within mHealth. Drawing on examples from other biomedical domains, we describe the positive impacts of sharing for three different types of resources and identify early efforts to encourage this behavior within mHealth. Thus, the insights offered through this and the other three articles in this issue can catalyze diverse activities to bring mHealth capabilities into clinical research and care.

## mHealth Connect workshop

Despite the growing body of literature on consumer-oriented mHealth devices, there is a paucity of strong evidence for their benefit^9^. Few applications have made the leap from prototype to routine use for research or clinical purposes. mHealth Connect (http://mobilize.stanford.edu/mhealthconnect/) was a workshop that brought together key stakeholder leaders across industry, clinical systems, and academia to collaboratively identify and overcome barriers to this translation. The workshop was launched in 2016 by two of the National Institutes of Health’s (NIH) Big Data to Knowledge (BD2K) Centers of Excellence–the Mobilize Center^11^ and the Mobile-Sensor-to-Knowledge Center (MD2K)^12^—in response to concerns voiced by many BD2K researchers that many commercial mobile devices and apps on the market are poorly validated, without compelling clinical use cases, and are opaque and restrictive about data sharing. mHealth Connect enabled discussions around these and other critical issues to take place with a balance of stakeholders at the table and seeded collaborations to advance the field. The three mHealth papers in this issue arise from those discussions and the needs identified during them.

## Scope of mHealth covered

While mHealth comprises a broad range of topics, as an outgrowth of two NIH Big Data to Knowledge Centers, mHealth Connect’s focus is on accelerating the use of data collected from mobile and wireless devices, such as wearable sensors, in clinical research and care. Because of the personal ubiquitous nature of mobile devices, the greatest new opportunity is in using mHealth to directly measure and improve patient health and health states outside the traditional confines of the hospital and clinic. The scope for this and the accompanying three papers thus excludes the following topics: (1) sensors and devices designed exclusively for the hospital or clinic setting and are intended solely to inform clinical decision-making (e.g., a Holter monitor, which would be excluded, versus AliveCor’s KardiaMobile device, which would be included), (2) strictly educational apps that are one-way channels for fixed media, (3) electronic health records (EHR) apps, and (4) apps for navigating the healthcare system (e.g., finding doctors, scheduling appointments) rather than for managing health or disease.

What these three papers do focus on are mobile apps and sensors used by patients in their daily lives to manage their health, with or without co-management by clinical team members or friends and family. These include devices measuring novel biomarkers, as well as consumer versions of traditional clinical equipment, such as blood pressure cuffs and spirometers, which an individual can use to collect measurements whenever and wherever they desire independent of clinical indications. The devices may be integrated into a clinical healthcare workflow, but they are not designed exclusively or primarily for that environment. The emphasis is on the availability of dynamic personalized data captured either passively or through active self-report, and the consequent value of this data for informing patient and clinician action to improve health and manage acute or chronic disease.

## Guidelines for developing and deploying mHealth solutions

Recent years have seen a rise in resources providing guidelines to evaluate mHealth solutions, including from the U.S. Food and Drug Administration (FDA)^13–16^. Evaluation criteria assess a broad range of factors, including adherence to privacy laws, data security, interoperability with existing infrastructure and workflows, cost, usability, and validity of the content or intervention. Nascent efforts, such as Express Scripts’ planned digital health formulary, a list of approved digital health technologies to guide consumers and payers, are emerging to reinforce these guidelines^17^. While efforts to increase rigor in the evaluation of mHealth solutions are still taking shape, many questions remain on best practices and frameworks for mHealth development upstream of final regulatory or formulary approval.

The Clinical Trials Transformation Initiative (CTTI) provides one of the more comprehensive sets of guidelines for developing a mobile device-based solution, including the development of novel endpoints from mobile device data and the design of protocols that use mobile devices for data capture^18^. CTTI’s guidelines are intended for the relatively controlled conditions and limited durations of clinical trials, and therefore, necessarily exclude considerations for broad-scale clinical deployment. Nonetheless, they provide a useful path for individuals launching mHealth endeavors in general. Below we introduce a collection of articles based on our series of mHealth Connect meetings that augment existing guidelines provided by CTTI and others^18–20^.

### Device selection for wellness, healthcare, and research applications

Regardless of the application, defining the target use case is critical for success. This definition is a fundamental tenet of many mHealth guidelines^18–20^, and it requires a process of user-centered design incorporating clinical, engineering, behavioral science, ethical, and disparities considerations (e.g., language, numeracy, literacy, and disabilities). All mHealth projects, even noncommercial ones, should have a clear business case detailing how continued use of the solution will be financially and logistically sustainable. The paper by Caulfield, et al. presents a framework for optimizing the match between sensors and classes of use cases, for refining the use case requirements, and then evaluating available devices against those requirements^21^.

### Analysis of digital biomarkers for predictive models and unique insights

Digital biomarkers are clinically meaningful measures derived from mobile and wearable devices that correlate with or predict disease states. They can be analogues of traditional clinical quantities, such as heart rate, or novel indicators of health states. The full impact of mHealth comes from simulation or predictive models that combine digital biomarkers potentially with other data sources. An example is the cStress model, which blends real-time data streams on heart rate, heart rate variability, and interbeat interval data to derive a probability of stress in a given 1-min time window^22^. Developed using MD2K’s Cerebral Cortex, a cloud infrastructure for big data analysis of high-volume high-frequency data streams^12^, cStress utilized a prospective approach and actively recruited participants to collect data for its development.

Data analysis and model building can also be done retrospectively on observational datasets to gain insights that are challenging to obtain through traditional studies. In some cases, these datasets contain upwards of hundreds of thousands of individuals, enabling analysis about health and behavior on an unprecedented scale^23,24^. While such datasets can be a windfall, they present their own set of unique challenges for obtaining reliable results. The paper by Hicks, et al. presents a set of best practices for analyzing these large-scale, observational digital biomarker datasets from commercial personal technologies^25^.

### Deploying mHealth solutions within clinical care

The necessity of a well-defined use case and business case becomes especially evident when it comes to the adoption and scaling of a mHealth solution. Is the mobile technology to be used by people with or without their clinicians? Is the intent to deploy locally in one care setting or to scale to global use? Particularly where clinician use is envisioned, integration into the clinical workflow is a prerequisite for adoption. To help guide expansion of mHealth technology into clinical care delivery, the paper by Smuck, et al. presents common factors driving successful utilization of wearables in the clinical care environment, as shown by two examples^26^.

## Resource sharing to accelerate mHealth adoption

These papers aim to increase the likelihood of mHealth projects to achieve their aims, whether that is integrating mHealth technologies into the clinical workflow or developing a model to accurately predict health outcomes from mHealth data. The recommendations are intended to advance the work of individual groups, but they also point to opportunities for collective efforts that would advance activities across the entire community. In particular, we highlight the impact of sharing digital resources. Echoing Hicks, et al., we encourage “sharing models, software, datasets, and other digital resources whenever possible”^25^. Below we describe three categories of shared resources that can accelerate mHealth’s leap to research and the clinics: raw and processed data from devices; software and models used to analyze and interpret data; and evaluation results. We call attention to the positive impact the sharing of such resources has had in other biomedical domains and highlight initial efforts to bring these practices to mHealth.

The benefits of sharing experimental data, software, and models are well-delineated: enhanced transparency, the ability to more rapidly and easily extend existing efforts, and decreased duplication of efforts^27^^,^^28^. Large biomedical datasets that have been established specifically as research resources, such as the UK Biobank and the Osteoarthritis Initiative, have demonstrated the value of sharing, having supported hundreds of published research studies^29^^,^^30^. Smaller datasets from independent research labs can also positively impact a field. Previously shared data have served as benchmarks for comparing algorithms and aided in the validation of new models^31–33^. And pooling these datasets, such as in the Enhancing Neuro Imaging Genetics through Meta-Analysis (ENIGMA) project, has increased the statistical power of analyses and led to discoveries that would not have been possible from a single dataset alone^34^.

Such capabilities are critical for advancing mHealth. Having readily accessible datasets, both large and small, will facilitate the development of new digital biomarkers or robust mHealth-based predictive models, such as those described by Hicks, et al. We are starting to see some organized efforts to promote data sharing among the mHealth community. The International Children’s Accelerometry Database (ICAD) has created a pooled dataset, similar to ENIGMA^35^. Vivli, a platform for sharing data from clinical trials including trials with digital biomarkers, was launched in 2018^36^, and SimTK, a repository for the biocomputation and movement communities, recently added support for the sharing of mobile and other experimental data^37^.

Although invaluable, shared data alone will not propel mHealth applications into routine research and clinical use. Software methods and models are needed to glean insights from the data and thus, it is just as important that they also be shared, ideally with an open-source license to encourage modification and reuse for new applications. The programming language Python is a testament to the power of open-source with over 100,000 community-developed extensions^38^ that make it a popular tool within bioinformatics and scientific computing. In biomechanics, researchers are sharing software for analyzing movement data within the open-source OpenSim simulation platform, extending the community’s ability to derive new insights^39^. While some mHealth software is being shared^40^^,^^41^, large, active communities have yet to develop around them. Initiatives, such as Open mHealth^42^ as well as Shimmer and Nextbridge Exchange’s industry-based open-source effort to share analysis tools for wearable sensor data^43^, may help change the culture.

Similar initiatives would be useful throughout the mHealth development process, including the sharing of evaluation results, for example when evaluating devices during study design, as described by Caulfield, et al.^21^, and also when developing a reimbursement model to implement wearable technology into patient care, as mentioned by Smuck, et al.^26^. If individuals made their evaluation results available for others to leverage, we could appreciably streamline these processes. The CTTI Feasibility Studies Database^44^ is a step towards this. The database compiles a list of devices, along with relevant evaluation criteria such as outcome measures and sample size, from publications examining the feasibility of mHealth in clinical trials. In a similar vein, the Digital Medicine Society provides a crowdsourced library of digital endpoints being used in industry-sponsored studies^45^. While there are some concerns about resource sharing—for example, potential misuse of shared resources and privacy breaches—technological and policy solutions can be implemented to mitigate them^46,47^. Compiling mHealth knowledge, data, and methods with such safeguards will accelerate the widespread adoption of mHealth for research and clinical care, and we urge individuals to contribute to such efforts.

## Conclusion

It has been 13 years since the first iPhone was released, and 11 years since the first FitBit. In the intervening years, smartphone adoption has skyrocketed, fitness bands and smartwatches are commonplace, and “mobile health” NIH grants have grown from tens per year to over 610 in 2019. It has been said that digital health is now at “the end of the beginning”^48^. The mHealth Connect events have highlighted ways to go beyond the beginning: develop cross-disciplinary collaborations, pay attention to purpose, and consider factors beyond the technology itself. The papers in this series are intended as a guide for mHealth’s journey ahead and highlight ways in which we can collectively accelerate our progress along the path to clinical research and care.
